# The cost of smile: how individual and organizational factors moderate the impact of emotional labor on work alienation via burnout

**DOI:** 10.3389/fpsyg.2025.1668413

**Published:** 2025-11-28

**Authors:** Engin Üngüren, Ömer Akgün Tekin, Hüseyin Avsallı

**Affiliations:** 1 Faculty of Economics and Administrative Sciences, Department of Business Administration, Alanya Alaaddin Keykubat University, Alanya/Antalya, Türkiye; 2 Manavgat Faculty of Tourism, Department of Gastronomy and Culinary Arts, Akdeniz University, Manavgat/Antalya, Türkiye

**Keywords:** emotional labor, burnout syndrome, work alienation, service orientation, perceived managerial support

## Abstract

**Introduction:**

In the tourism industry, frontline employees are exposed to intense customer interactions that require emotional labor. It is critically important to understand the effects of surface acting and deep acting strategies—performed within the framework of organizational display rules—on adverse psychological consequences such as burnout and work alienation. Building on the Conservation of Resources (COR) and Job Demands–Resources (JD-R) theories, this study examines the mechanisms and boundary conditions of this negative cycle. This study examines an integrated model examining the mediating role of burnout in the relationship between emotional labor strategies (surface and deep acting) and work alienation, and the moderating (buffering) roles of service orientation (as a personal resource) and managerial support (as an organizational resource) in this process.

**Method:**

Data were collected through random sampling from 1,252 employees working in five-star hotels located in the Alanya and Manavgat regions of Türkiye and analyzed using Partial Least Squares Structural Equation Modeling (PLS-SEM).

**Results:**

The findings revealed that surface acting significantly increased both burnout and work alienation, whereas deep acting significantly mitigated these adverse outcomes. Burnout was identified as a partial mediator in the relationship between emotional labor strategies and work alienation. A key finding was the significant buffering effect of both service orientation and managerial support on the relationship between emotional labor and burnout. These resources substantially weakened the positive effect of surface acting on burnout, thereby reducing its detrimental consequences.

**Discussion:**

The results demonstrate that the negative psychological costs of emotional labor can be effectively managed through individual and organizational resources. Theoretically, integrating multi-level resources within a unified model provides a more nuanced understanding for the emotional labor literature. Practically, the findings suggest that hospitality organizations should prioritize selecting service-oriented individuals during recruitment and invest in training programs that foster supportive leadership behaviors among managers.

## 1 Introduction

Employees' attitudes and behaviors during service delivery in the hospitality industry constitute one of the fundamental determinants of organizational success (Karatepe and Aleshinloye, 2009; Pahi et al., 2022; Shani et al., 2014; Tsai, 2009). Frontline employees specifically, who are in direct contact with customers, play important roles in shaping the organization's perception of service quality as well as customer satisfaction (Wang, 2020). To positively enhance this effect in alignment with organizational goals, hospitality businesses often set up “display rules” that govern employees' emotions, gestures, mimics, tones of voice and manners of expression (Chehab et al., 2021; Cheng et al., 2025; Kaya and Serçeoglu, 2013; Lee and Ok, 2015). Display rules offer a framework to steer employees' interpersonal relationships and improve organizational efficiency (Nguyen et al., 2023; Chen et al., 2012). Employees, who abide by such rules and effectively regulate their emotions, can positively impact customers' perception of service quality (Cheng et al., 2022; Medler-Liraz and Seger-Guttmann, 2015), level of satisfaction (Hur et al., 2013) and organizational commitment (Wang, 2019). In this context, employees' adherence to display rules and the development of emotional regulation skills directly influence the customer experience and, in turn, the success of the organization.

In order for display rules to be effectively implemented, employees are required to perform emotional labor. Emotional labor is defined as the expression of emotions for pay in line with organizational goals in seminal studies (Hochschild, 1979, 1983). In contemporary research, however, emotional labor is considered a process in which employees regulate their emotions within the framework of display rules (Hong and Kim, 2023) or manage their emotions to meet job requirements (Hu et al., 2023). Existing studies highlight the critical role of emotional labor often within the context of the organization's success (Cheng et al., 2025; Lee and Hwang, 2016; Tsai, 2009; Xu et al., 2020).

Emotional labor regulations typically operate through two strategies: deep acting (transforming inner feelings to align with the displayed emotion) and surface acting (displaying emotions that are not genuinely felt, in a “fake” manner; Lu et al., 2019). However, employees' expression of emotions they do not genuinely feel or suppression of emotions they actually experience, can lead to increased levels of emotional stress (Ashforth and Humphrey, 1993; Grandey, 2000; Lim et al., 2024; Mann and Cowburn, 2005; Peart et al., 2012; Pugliesi, 1999; Tian et al., 2024; Yikilmaz et al., 2024). The mismatch between felt and expressed emotion is referred to as “emotional dissonance” and poses a threat to an individual's psychological integrity. Over time, chronic emotional dissonance may lead to an internal conflict between the employee's “authentic self” and “work self” (Ashforth and Humphrey, 1993; Cha et al., 2019; Chang, 2020; Jansz and Timmers, 2002; Johannessen et al., 2025; Melita, 2023; O'Brien and Linehan, 2019). Such dissonance can pave the way for burnout by increasing psychological tension (Chang, 2013; Chen et al., 2024; Grandey, 2000; Jeung et al., 2018; Kariou et al., 2021; Landay et al., 2022; Nguyen and Stinglhamber, 2021; Wharton, 1999; Zapf, 2002). Burnout is defined as the typical reaction to chronic professional stress and is observed more often in environments where emotional demand is especially high and organizational support is low (Brotheridge and Grandey, 2002; Dudina and Martinsone, 2025; Maslach and Jackson, 1981; Yanbei et al., 2023). The risk tends to increase when feelings of autonomy and competence are low (Öztürk Çiftçi, 2021). Burnout may strengthen the tendency toward work alienation, accompanied by experiences such as detachment, helplessness, and hopelessness (Billeter-Koponen and Fredén, 2005; Çiçek Korkmaz and Torlak, 2024; Gülşen and Senol, 2025; Mottaz, 1981; Powell, 1994; Pilette and Olio, 1980; Schaufeli and Buunk, 2002; Zhang et al., 2023). Thus, burnout is not only a consequence of emotional labor but may also serve as a potential trigger for work alienation (Karayman, 2024; Zhong and Bu, 2025).

This study adopts the Conservation of Resources (COR) Theory (Hobfoll, 2001) and the Job Demands–Resources (JD-R) Model (Bakker and Demerouti, 2017) as its main theoretical frameworks to explain and prevent these negative dynamics. COR predicts that under insufficient resources or excessive demands, individuals may rapidly deplete their emotional and physical reserves, leading to negative outcomes (Hobfoll, 2001; Chen et al., 2024; Clarke et al., 2024; Peng and Li, 2023). JD-R, on the other hand, approaches work environments as a combination of demands and resources and emphasizes the buffering effect of resources on the tension created by demands (Chen et al., 2023; Demerouti and Bakker, 2022). JD-R suggests that the presence of workplace resources helps employees cope with demanding job requirements and maintain their wellbeing (Chen et al., 2023; Demerouti and Bakker, 2022). This study examines resources on individual and organizational levels. Service orientation is a personal resource, providing employees with emotional tools to cope with the daily stress (Abrian et al., 2024; Cheng et al., 2022; Gursoy et al., 2011). It has been shown that individuals with higher levels of service orientation employ deep acting strategy more often and therefore are emotionally more resilient (Bakar et al., 2020). Moreover, employees with higher levels of service orientation tend to quickly adapt to customer expectations, establish positive interactions (Yi et al., 2022) and respond to demands enthusiastically (Xu et al., 2020). When the emotional requirement of the job is in parallel with employees' self-images, emotional labor becomes easier to manage (Brotheridge and Grandey, 2002; Isenbarger and Zembylas, 2006; Talaifar and Swann, 2017; Yin et al., 2017). Accordingly, employees with individual resources such as service orientation, are more likely to be resilient toward burnout and work alienation in addition to being less likely to experience conflict. Described as the social and emotional support employees receive from their managers, managerial support is considered to be a significant organizational resource. Managerial support can aid in alleviating the emotional load of the job (Bourgoin Boucher et al., 2024; Cheng et al., 2025; Pohl et al., 2023; Yang et al., 2016). Studies have also shown that sufficient managerial support can improve resilience against burnout in employees (Blanch and Aluja, 2012; Chong et al., 2024; Deery et al., 2002; Dobnik and Lorber, 2023; Grobelna, 2021; Karatepe and Kilic, 2015; Luo et al., 2022; Shahwan et al., 2024; Sterud et al., 2011).

The fact that the attitudes and behaviors of employees in the hospitality sector during service delivery are the key to organizational success demonstrates that this field is of critical importance for research (Gursoy et al., 2011; Kim et al., 2023; Pujol-Cols et al., 2023; Shani et al., 2014). However, there remains a need for up-to-date findings that can guide industry professionals in understanding the potential adverse effects of emotional labor and how these effects can be mitigated. To address these gaps and provide empirically grounded strategies, this study is guided by the following central research questions:

- What are the differential effects of emotional labor strategies (deep acting vs. surface acting) on job burnout and work alienation?
- Does job burnout mediate the relationship between emotional labor strategies and work alienation?
- Do service orientation (as a personal resource) and managerial support (as an organizational resource) moderate the relationship between emotional labor strategies and job burnout?
- Do these personal and organizational resources also moderate the indirect effect of emotional labor strategies on work alienation *through* job burnout?

Therefore, this study is expected to contribute to the existing body of knowledge in the following ways: (i) clarifying emotional labor by distinguishing it as strategy-based and by linking emotional labor with work alienation through burnout; (ii) testing the moderating role of both an individual (service orientation) and an organizational (managerial support) resource in the same model and providing an integrative perspective on “for whom” and “under which conditions” the negative outcomes of emotional labor are effective; (iii) providing a theoretical foundation that models both the resource loss spiral and the resource buffer processes by integrating the COR and JD-R theories; (iv) proposing empirically grounded strategies for addressing common issues such as burnout and alienation in the sector by guiding the development of a sustainable human resource management approach that safeguards employee wellbeing. Accordingly, the study aims to make an original theoretical and practical contribution to the literature by clarifying when, how, and for whom emotional labor produces detrimental or protective outcomes in the context of the hospitality sector.

## 2 Literature review

### 2.1 The effect of emotional labor on burnout

Within the framework of one of its theoretical foundations, the Conservation of Resources (COR) theory, this study integrates the strategy-based distinction in emotional labor theory (deep vs. surface acting) and examines the differentiated effects of these strategies on job burnout and, through burnout, on work alienation, among frontline employees in the hospitality sector. According to COR, individuals strive to protect, maintain, and enhance their valuable resources such as psychological energy, self-efficacy, and social support (Hobfoll, 1989). When resources are lost or the balance between resource investment and return is significantly disrupted, stress emerges; if appropriate and timely resource replenishment cannot be ensured, the process evolves into burnout (Sun et al., 2025). The effects of emotional labor on the psychological wellbeing of service-sector employees continue to attract scholarly attention (Lei and Kuok, 2025; Yao et al., 2025; Yang and Chen, 2021; Uysal et al., 2020). In this context, the hospitality sector, characterized by intensive and continuous customer relations, provides a particularly appropriate setting to investigate why employees' efforts to regulate their emotions are not sustained anymore and lead to burnout.

Hochschild (1979), the pioneer of the emotional labor concept, defined it as a form of labor sold for a wage and emphasized its exchange value. In contemporary research, however, the concept is reflected in a more process-oriented way and is addressed as the regulation of emotions within the framework of display rules (Hong and Kim, 2023) or the management of emotions to meet job requirements (Hu et al., 2023). Emotional labor continues to be examined at two main strategy levels: surface acting and deep acting. Surface acting refers to a deliberate, imitative effort by employees to display emotions they do not genuinely feel or to suppress those they actually experience (Nguyen and Stinglhamber, 2021). Deep acting, on the other hand, involves employees' cognitive efforts to internalize and genuinely feel the emotions they are expected to display, providing a more authentic emotional experience (Diefendorff et al., 2005).

Although there is not yet a widely established consensus in existing studies on emotional labor (Guo, 2023; Saei et al., 2024; Yin, 2023; Yin et al., 2023), findings suggest that surface acting tends to have a positive association with burnout, while deep acting is associated with a negative one. From the COR perspective, surface acting produces a self-role incongruence that requires constant cognitive control and emotional suppression (Jalilzadeh et al., 2024). This process accelerates the depletion of employees' psychological resources (Grandey, 2000). The chronic persistence of this incongruence may lead to the exhaustion of emotional resources and ultimately to increased burnout (Chen and Wei, 2023; Hobfoll et al., 2018; Saei et al., 2024). In contrast, deep acting is based on aligning felt emotions as closely as possible with display rules (Teoh et al., 2019). The reduction in the discrepancy between felt and displayed emotion lowers the cost of self-regulation and the strain on psychological resources, hence carrying the potential to reduce burnout levels. Numerous studies also have reported significant relationships between emotional labor and burnout (Amissah et al., 2021; Choi and Kim, 2015; De Vries et al., 2022; Ha et al., 2021; Hur et al., 2013; Lv et al., 2012; Popucza et al., 2025; Zhang et al., 2024; Zhang and Zhu, 2008). Therefore, surface acting can be argued to trigger loss spirals, whereas deep acting activates protective/resource-gaining pathways.

Empirical studies conducted in the hospitality sector support this theoretical framework. Kim (2008) found that surface acting increased burnout among hotel employees, while no significant relationship was observed between deep acting and burnout. Studies on hotel employees and flight attendants conducted by Lv et al. (2012) and Hur et al. (2013) reported that surface acting heightened emotional exhaustion, whereas deep acting reduced this effect. Similarly, Amissah et al. (2021) found that surface acting increased emotional exhaustion, while deep acting decreased it among frontline employees in the hospitality sector. Jeung et al. (2018), in their meta-analytic investigation, demonstrated that emotional labor is a core work stressor that leads to burnout. Satyanarayana and Shanker (2012), on the other hand, showed that emotional labor performance among hotel employees increases burnout. Building on these theoretical and empirical foundations, the first hypotheses of the research were formulated as follows.

**H1a:** Deep acting affects burnout negatively.
**H1b:** Surface acting affects burnout positively.

### 2.2 The effect of burnout on work alienation

In general terms, alienation refers to a state in which an individual feels disconnected from their surroundings, is unable to establish a meaningful connection with their environment, or experiences a loss of that connection (Seeman, 1959). Alienation has been examined in various contexts across multiple disciplines, including sociology, psychology, philosophy, and theology (Fromm, 1956; Horowitz, 1966; Marx, 1981; Oliver, 1989; Xiaoyan, 2017). In this study, the concept of “alienation” is addressed within the scope of “work alienation” as defined by Mottaz (1981), which refers to employees' feelings of powerlessness and meaninglessness in relation to the work environment. Accordingly, work alienation can be defined as a condition in which the employee becomes detached from their job and shows a lack of engagement with their role in the workplace due to factors stemming from work conditions (Hirschfeld and Feild, 2000). Work alienation also encompasses the negative emotions that arise when an individual is unable to align with professional norms (Aiken and Hage, 1966). This refers to feelings of dissatisfaction or detachment related to the employee's role, the way tasks are carried out, and the outcomes of their work.

One of the leading authorities in the relevant literature on alienation, Seeman (2023, p. 784) conceptualized alienation as a multidimensional construct composed of powerlessness, meaninglessness, normlessness, social isolation, and self-estrangement. In this context, work alienation is most commonly explained through the individual's sense of powerlessness. Defined as “a low expectancy that one's own behavior can control the occurrence of personal and social rewards,” this sense of powerlessness (Seeman, 1972, p. 112) not only creates a fertile ground for the emergence of work alienation (Marx, 2001; Sharma, 2007; Vanheule and Verhaeghe, 2004) but is also closely linked to burnout. After all, emotional states such as helplessness, hopelessness, and powerlessness are frequently observed in employees experiencing burnout (Billeter-Koponen and Fredén, 2005; Pilette and Olio, 1980; Schaufeli and Buunk, 2002). In this context, various existing studies (Akar, 2018; Conway et al., 2018; Inandi and Büyüközkan, 2022; Khan et al., 2019; Öztürk Çiftçi, 2021; Powell, 1994; Taboli, 2015; Usman et al., 2020; Yu et al., 2021) further revealed positive links between burnout and work alienation. Based on this information in the literature, we propose the second hypothesis for the research study:

**H**_**2**_**:** Burnout affects work alienation positively.

### 2.3 The moderating role of service orientation and managerial support in the relationship between emotional labor and burnout

JD-R approaches all work environments through two main components. The first one, job demands, refers to aspects of the job that require sustained physiological or psychological effort and therefore generate strain. The second, job resources, comprises factors that facilitate coping with demands, enhance goal orientation and motivation, and enable learning and recovery (Tummers and Bakker, 2021). Within the context of the hospitality sector, display rules and intense customer contact create distinctively high demands, as they require employees to engage in continuous self-regulation. At this point, the JD-R model's emphasis on resources becomes critical. These resources can operate at different levels. This study positions two critical protective mechanisms at its core, distinguished by their source: service orientation (as an individual-level personal resource) and managerial support (as an organizational-level job resource). These resources are predicted to reduce the likelihood of adverse organizational outcomes by enabling employees to cope more effectively with emotional demands. The specific buffering roles of each resource level are detailed below.

#### 2.3.1 The moderating role of service orientation (individual level)

Service orientation is an individual-level personal resource, conceptualized as a core personality trait that enables employees to act kindly, considerately, cooperatively, and helpfully toward customers and other stakeholders, and motivates them to be willing and enthusiastic in delivering service (Hogan et al., 1984). Individuals with a strong service orientation derive satisfaction from helping others and possess a high level of intrinsic motivation to do so (Chan and Wan, 2012). Therefore, when faced with situations that require emotional labor, individuals with a high level of service orientation are less likely to experience burnout (Bakar et al., 2020; Huang et al., 2019; Lee and Ok, 2015; Piaralal et al., 2016). Underlying this phenomenon are findings in the literature suggesting that personality traits such as *openness, extraversion, conscientiousness, agreeableness, and emotional stability* enhance individuals' resilience to burnout (Ghorpade et al., 2007; Madnawat and Mehta, 2012; Zellars et al., 2000). Service orientation is also positively associated with these personality traits (Donavan, 1999; Köşker et al., 2019; Serçeoglu, 2013; Tekin and Kalkan, 2017). Employees with high service orientation may find satisfaction in helping others, which enables them to replenish or preserve their resources (Brown et al., 2002).

Employees with high service orientation are not only inclined to help others but also derive deep personal satisfaction from such activities. The intrinsic fulfillment they gain from helping others may help them navigate the emotional demands of their roles more effectively. As a result, service orientation can function as a buffer against burnout (Babakus et al., 2011; Babakus and Yavas, 2012; Donavan et al., 2004; Wu and Shie, 2017). On the other hand, employees with low service orientation may face a more intense and demanding emotional labor process in order to comply with organizational display rules. This can quickly deplete their emotional and cognitive resources, triggering burnout and ultimately increasing their intention to leave the job (Babakus et al., 2011).

Therefore, service orientation may play a significant role in preventing burnout in jobs that require emotional labor. Bakar et al. (2020) found that employees with high service orientation experience fewer emotionally taxing feelings during service encounters, while Çoban and Aytemiz Seymen (2019) noted that surface acting can lead to negative outcomes in customer relations by suppressing emotions. Bakar et al. (2020) further observed that employees with strong service orientation are more likely to engage in deep acting, which reduces emotional exhaustion. Yoo et al. (2015), on the other hand, identified a negative relationship between service orientation and burnout. Therefore, we propose that employees with high service orientation are better equipped to manage emotionally demanding situations and can thus mitigate the negative impact of emotional labor on burnout. Based on this reasoning, the third hypothesis of the study is formulated as follows:

**H3a:** Service orientation plays a moderating role in the effect of surface acting on burnout.
**H3b:** Service orientation plays a moderating role in the effect of deep acting on burnout.

#### 2.3.2 The moderating role of managerial support (organizational level)

Distinct from individual-level resources, job resources are organizational-level factors provided by the work environment. Managerial support is considered a key job resource in fostering a supportive atmosphere in the workplace, enhancing employee motivation (Hasnat Bhatti et al., 2023), and contributing to organizational success (Chayomchai, 2020). Such support is provided to help employees cope with challenges and to offer them the encouragement they need (Farrukh et al., 2019). Considered a component of organizational support, managerial support (Thatcher et al., 2007) encompasses the psychological and physical assistance that supervisors offer to their employees (Basu and Green, 1997).

Existing studies have shown that managerial support produces numerous positive outcomes for both employees and organizations. For example, it contributes to employee skill development and increased intrinsic motivation (Smith-Jentsch et al., 2001), strengthens organizational citizenship behavior (Mayfield and Mayfield, 2014), and enhances job satisfaction and organizational commitment (Pelenk, 2020; Waldan, 2020). Surface acting, which demands the highest level of uncompensated emotional output, is particularly sensitive to support mechanisms (Sun et al., 2025). Cheng et al. (2025) found that empowering leadership encourages employees to adopt more deep acting strategies while reducing surface acting behaviors. Managerial support also helps improve employee performance (Sürücü et al., 2022) and reduces turnover intentions, hence contributing to the organization's competitive advantage (Gordon et al., 2019). From the perspective of customer satisfaction and loyalty, managerial support is reported to have indirect yet critical effects on service quality (Grobelna, 2021), customer satisfaction (Susskind et al., 2018), and customer loyalty (Maria Stock et al., 2017).

On the other hand, a low level of perceived managerial support can lead to various negative outcomes for both organizations and employees. One such outcome is burnout, which is frequently observed among frontline employees in the hospitality industry (Walters and Raybould, 2007). When this organizational resource is absent, employees are deprived of a critical coping mechanism for managing stress (Birkeland et al., 2017). As a consequence, the inability to relieve stress in a sustainable manner may create conditions that give way to the development of burnout (Maslach et al., 2001). Alternatively, strengthening managerial support within the organization is considered a promising approach to reducing the effects of burnout (Blanch and Aluja, 2012; Deery et al., 2002). Indeed, previous studies (Altaş et al., 2024; Blanch and Aluja, 2012; Charoensukmongkol et al., 2016; Choi et al., 2012; Desrumaux et al., 2018; Elçi et al., 2018; Gibson et al., 2009; Grobelna, 2021; Khatatbeh et al., 2020; Qotimah et al., 2021; Weigl et al., 2016) have shown the existence of negative relationships between managerial support and burnout. Taken together, these findings suggest that the impact of emotional labor on burnout may be mitigated by perceived managerial support. Therefore, we propose the following hypotheses in our study:

**H4a:** Managerial support plays a moderating role in the effect of surface acting on burnout.
**H4b:** Managerial support plays a moderating role in the effect of deep acting on burnout.

### 2.4 The effect of emotional labor on work alienation

The requirement for employees to manage their emotional states throughout work processes has a significant impact on their performance and may lead to alienation from their own emotional experiences. This highlights the challenges and complications of the concept of emotional labor. Hochschild (1983) approached this issue from a wider perspective, suggesting that employees may lose control over their emotional regulation mechanisms as time goes by, which can lead to a loss of control over other aspects of their work and ultimately result in a general state of work alienation. The negative relationship between deep acting and work alienation was observed in the study conducted with hotel employees by Yildirim and Türker (2018). Likewise, Kurt et al. (2019), in their research on tourism sector workers, found a negative correlation between deep acting and alienation, reporting that employees who engaged in deep acting experienced lower levels of alienation. Tokmak (2014), in a study on logistics employees, concluded that work alienation can result from emotional labor. Zaganjori (2016) also found a positive relationship between emotional labor and alienation, drawing on data from travel agency staff. Yildiz (2017), in a study with flight attendants, reported that the suppression of emotions and the resulting emotional dissonance were positively associated with alienation in its dimensions of meaninglessness, powerlessness, and detachment. Similarly, Ahmed (2022) identified emotional labor as a strong predictor of work alienation among hotel employees. During the surface acting process, employees are compelled to display emotions they do not genuinely feel while suppressing their authentic emotional states. Over time, this dissonance leads to a growing sense of alienation from their work (Mastracci and Adams, 2018). This is because the loss of control over one's emotional expression in the workplace transforms emotional labor into “alienated labor” (Garfield, 1980). The persistent demand to simulate false emotions in order to meet occupational expectations is found to lead to a sense of meaninglessness and ultimately to self-alienation. Based on this review, we propose the following fifth hypothesis for the research study:

**H5a:** Surface acting has a positive effect on work alienation.
**H5b:** Deep acting has a negative effect on work alienation.

### 2.5 The mediating role of burnout in the relationship between emotional labor and work alienation

Since Hochschild's (1979) pioneering work, emotional labor has been emphasized as a process that depletes individual resources and may lead to various negative outcomes (Lv et al., 2023; Sun et al., 2025). Emotional labor entails employees' continuous confrontation with job demands that require emotional regulation. From the COR perspective (Hobfoll, 2001), such demands constitute a critical stressor that consumes individual emotional and cognitive resources. Surface acting involves suppressing genuine emotions and displaying false ones, and therefore requires continuous self-regulation, resulting in resource depletion (Lei and Kuok, 2025; Lyddy et al., 2021; Yin et al., 2019; Zhai et al., 2025). This effort initially manifests as heightened job stress (Choi et al., 2019; Jung and Yoon, 2014; Pugliesi, 1999; Sohn et al., 2018; Yoon and Kim, 2011). When chronic, surface acting becomes the primary mechanism triggering burnout (Maslach et al., 2001; Schaufeli and Enzmann, 1998; Chiang and Liu, 2017; Sun et al., 2025; Smith et al., 2025).

Burnout, on the other hand, erodes employees' sense of meaning and control over their work. In turn, it causes detachment, helplessness, and a loss of significance, which reinforces work alienation (Schaufeli and Buunk, 2002; Zhang et al., 2023). Alienated employees are unable to control their work, lose a sense of purpose, and become estranged from their roles (Mottaz, 1981). In this regard, the effect of emotional labor on work alienation can be explained through the mediating role of burnout. This mediating relationship is consistent with the COR theory (Hobfoll et al., 2018). COR posits that high-demand processes such as emotional labor rapidly deplete personal resources, and this depletion results in secondary losses such as alienation through burnout. Within the JD–R framework (Bakker and Demerouti, 2017), emotional labor can be conceptualized as a job demand, burnout as its negative outcome, and work alienation as the ultimate consequence. Employees who experience exhaustion in the face of high job demands may sever their emotional and cognitive ties with their work, leading to negative job attitudes (Chen et al., 2024; Saei et al., 2024; Santos and De Jesus, 2020). Sun et al. (2025), revealed that surface acting significantly predicts turnover intention, both directly and indirectly, through burnout.

Deep acting minimizes emotional dissonance. This strategy enhances employees' sense of accomplishment by aligning the internal and external expression of emotions. When employees autonomously embrace their work, perceiving it as part of their identity without external pressure, they tend to feel happier and more willing to perform their duties, which serves as a protective factor against emotional exhaustion (Chen et al., 2019; Sulistiawan et al., 2022). According to COR, employees with sufficient resources are more likely to engage in behaviors that preserve or generate additional resources (Cheng et al., 2025). Deep acting has been observed to significantly reduce emotional dissonance and the risk of burnout (Pinkawa and Dörfel, 2024). Deep acting also negatively influences burnout, and the reduction in burnout levels, in turn, decreases alienation. Zhang et al. (2025) found that deep acting enhances career resilience through mindfulness, which consequently reduces burnout. Based on this information in the literature, we propose the following hypotheses for the research study:

**H6a:** Burnout plays a mediating role in the effect of deep acting on work alienation.
**H6b:** Burnout plays a mediating role in the effect of surface acting on work alienation.

### 2.6 The moderating roles of managerial support and service orientation in the indirect effect of emotional labor on work alienation through burnout

Although the literature suggests that deep acting has fewer negative psychological effects on employees compared to surface acting, even deep acting can increase levels of burnout when performed under high and sustained job demands (Grandey, 2000; Saei et al., 2024). Surface acting has a stronger potential to trigger burnout more prominently by extending the mismatch between felt and expressed emotions (Hochschild, 1983; Maslach et al., 2001). Both strategies deplete employees' emotional and psychological resources, increasing the risk of burnout at varying levels. As burnout increases, feelings of detachment and meaninglessness grow stronger, thus heightening the tendency toward work alienation (Maslach et al., 2001; Mottaz, 1981; Schaufeli and Buunk, 2002). In this context, managerial support is considered a critical organizational resource for mitigating the negative outcomes of emotional labor (Duke et al., 2009). It not only addresses employees' emotional needs but also includes instrumental forms of support such as guidance and practical assistance in work processes (Blanch and Aluja, 2012; Somech and Drach-Zahavy, 2013). This support can weaken the process leading from burnout to work alienation, enabling employees to better manage the strain resulting from the demands of emotional labor (Charoensukmongkol et al., 2016; Desrumaux et al., 2018; Weigl et al., 2016). Li et al. (2021), reported that psychological empowerment can support frontline employees in actively modifying their emotions and behaviors through deep acting rather than surface acting. Especially in service sectors where face-to-face interaction with customers is frequent, the emotional understanding and support provided by managers play an effective role in reducing the psychological burden arising from employees' efforts to comply with emotional display rules (Somech and Drach-Zahavy, 2013). Based on these explanations, the following two hypotheses are proposed for the research:

**H7a:** Managerial support plays a moderating role in the indirect effect of deep acting on work alienation through burnout.
**H7b:** Managerial support moderates the indirect effect of surface acting on work alienation through burnout.

The literature indicates that individuals with high service orientation express themselves more effectively in customer interactions, experience lower levels of stress, and are particularly more successful in managing the emotional labor process (Donavan, 1999; Köşker et al., 2019). This personality trait also enhances the alignment between an individual's self-concept and the roles required by the job (Swann et al., 1987), which can in turn help alleviate mechanisms leading to burnout and work alienation. *Employees with high service orientation* view providing service and helping others as “valuable” (Köşker et al., 2019) and are hence more likely to tolerate negative emotional strain (Donavan, 1999). The intrinsic satisfaction they derive from interacting with people can partially reduce the impact of emotional dissonance experienced during emotional labor processes (Brown et al., 2002) helping to keep burnout levels under control and thereby lowering the likelihood of work alienation. Within this framework, the following two hypotheses are proposed:

**H7c:** Service orientation moderates the indirect effect of deep acting on work alienation through burnout.
**H7d:** Service orientation moderates the indirect effect of surface acting on work alienation through burnout.

## 3 Methods

### 3.1 Sample and procedure

Within the scope of this research study, data was collected from employees working in the frontline departments of four- and five-star hotels in the Alanya and Manavgat regions of Türkiye. Respondent selection criteria were designated to include employees who are in direct and continuous interaction with customers. Accordingly, the sample consisted of full-time employees working in guest relations, front office, food and beverage, and animation departments who had at least 6 months of work experience (including internships). Managerial positions, office staff, and support personnel without direct customer contact were excluded from the study. This way, an appropriate participant profile was established to achieve one of the main objectives of the research—understanding perceptions of managerial support and its relationship with organizational cynicism. These criteria were applied to ensure that respondents reliably reflected attitudes based on sectoral experience and customer interaction. In addition, participation in the study was voluntary; at the beginning of the questionnaire, it was clearly stated that participation was voluntary, that respondents could withdraw from the study at any stage, and that all responses would remain completely anonymous. The main sampling framework was developed using the “Ministry-Certified Facilities” list, which is regularly updated by the General Directorate of Investments and Enterprises under the Ministry of Culture and Tourism of the Republic of Türkiye. According to this list, there are 101 five-star hotels in Alanya and 165 in Manavgat (TMCT, 2025). The sample included employees from 85 hotels, who were randomly selected from within the population. To ensure an unbiased data collection process, the researchers employed a simple random sampling technique and selected hotels using a random number generator. During sampling, 37 hotels were selected from Alanya and 48 from Manavgat, resulting in a balanced dataset across the two regions. Data were collected between April and May 2025. Prior to data collection, the researchers obtained ethical approval from the relevant institutional review board (Akdeniz University—Social and Human Sciences Scientific Research and Publication Ethics Committee, Permission document number: E-55578142-050.99-1096316). During the data collection process, the researchers contacted the human resources managers and general managers of each hotel, providing detailed information about the scope and purpose of the study. The scientific nature of the research and the ethical approval documentation were shared, and consent to participate was obtained from the hotel administrations. A team of 11 surveyors worked alongside the researchers for the administration of the questionnaires. The primary criterion for selecting surveyors was that they had previously served as field interviewers in at least one scientific study. This prior experience was considered essential to ensure effective communication with participants and to ensure data quality. Before data collection, the surveyors participated in a 2-day training program organized by the researcher, consisting of 4 h per day. The training covered the purpose of the study, the content of the questionnaire items, ethical principles, procedures for introducing the study to participants, data confidentiality, and questionnaire administration techniques in detail. In addition, role-playing exercises were conducted to practice responding to potential respondent questions and managing the data collection process. Questionnaires were administered through face-to-face interviews with participants in settings approved by hotel management, such as break areas, cafeterias, or vacant meeting rooms deemed convenient for respondents. The survey sessions were conducted during employee breaks or at the beginning and end of work shifts, as coordinated by the human resources departments. In this study, the sample size was determined through an a priori power analysis using G^*^Power 3.1.9.7 software (α = 0.05, Power = 0.95, *f*^2^ = 0.05). The analysis revealed a minimum sample requirement of 402 participants. Following the recommendations of Ringle et al. (2014) for increasing sample size to achieve more stable and generalizable model estimations, the target number was increased to approximately three times this minimum threshold. A total of 1,286 questionnaires were collected; however, 34 were excluded from the analysis due to missing or erroneous data. As a result, 1,252 valid questionnaire forms were included in the analysis.

For the Turkish adaptation of the surface acting, work-related burnout, managerial support, customer-oriented service behavior, and work alienation scales used in this study, the back-translation method proposed by Brislin (1976) was employed. In the first stage, the original English versions of the scales were translated into Turkish by an expert fluent in both the source language (English) and the target language (Turkish). In the second stage, the resulting Turkish versions were given to another independent expert, who retranslated them into English. In the third stage, the back-translated English items were systematically compared with the original English items of the scales, and their semantic equivalence was evaluated. Based on this assessment, the Turkish items produced in the initial translation stage were accepted as the final scale items. Before the main survey, a pilot test was conducted with 30 participants to verify the clarity and comprehensibility of the items. The pilot results indicated that the items were clear and easily understood; therefore, no further revisions to the scales were necessary.

To minimize common method bias (CMB), several procedural and statistical control measures recommended by Podsakoff et al. (2003) were employed in this research. As part of these measures, the purpose of the study was clearly communicated to participants, and it was emphasized that participation was entirely voluntary. Participants were informed that they could withdraw from the study at any time without providing a reason. Anonymity and confidentiality were assured, and it was explicitly stated that the information provided would not be shared with third parties. The data collection instruments were designed in a way that excluded any questions that could reveal the identity of the respondents. This approach aimed to minimize the likelihood that participants would modify their answers based on social desirability or perceived expectations. In addition, the scale items were not grouped by variable; instead, they were presented in a mixed order to reduce potential bias that could result from consistent response patterns or common method variance. To determine whether common method bias (CMB) posed a problem, Harman's single-factor approach was adopted as one of the statistical techniques. Within the scope of Harman's Single-Factor Test, all measurement items were subjected to an exploratory factor analysis (EFA) without rotation. The results indicated that a single factor accounted for 27.9% of the total variance. Since this value is below the 50% threshold, it was concluded that common method bias did not constitute a significant concern in this research study (Podsakoff and Organ, 1986).

### 3.2 Measurement

The variables used in this study were measured using multi-item scales that had previously been tested for validity and reliability. All scales employed a five-point Likert-type format, ranging from 1 = Never/Strongly Disagree to 5 = Always/Strongly Agree.

#### 3.2.1 Surface acting

The surface acting subscale of the Hospitality Emotional Labor Scale developed by Chu and Murrmann (2006) was used to measure the level of surface emotional display among hotel employees. The scale consists of six items, and participants were asked to respond to each statement using a five-point Likert scale. Higher scores on the scale indicate that the employee outwardly expresses emotions that are not genuinely felt, in line with organizational expectations, reflecting the use of a surface acting strategy in the emotional labor process.

#### 3.2.2 Deep acting

Deep acting was measured using a four-item scale developed by Basim and Begenirbaş (2012). Participants evaluated statements regarding the extent to which they internalize and genuinely express their emotions, using a five-point Likert scale ranging from 1 to 5. This scale captures how employees regulate their internal emotional states during service delivery.

#### 3.2.3 Work-related burnout

The mediating variable in the research model, job burnout, was measured using a seven-item scale developed by Kristensen et al. (2005), which assesses individuals' levels of physical and psychological exhaustion related to their work. The items were scored on a five-point Likert scale. Higher scores indicate a higher perceived level of burnout, while lower scores reflect lower levels of perceived burnout.

#### 3.2.4 Managerial support

Managerial support, one of the moderating variables in the study, was measured using a six-item scale adapted by Zhang and Wang (2021). This scale assesses participants' perceptions of the level of support they receive from their supervisors and is structured in a five-point Likert format.

#### 3.2.5 Customer-oriented service behavior

The other moderating variable, customer-oriented service behavior, was measured using a five-item scale developed by Bettencourt and Brown (1997). This scale assesses employees' voluntary behaviors aimed at understanding customer needs, demonstrating empathy, and enhancing service quality. Participants scored the items on a scale ranging from 1 = Strongly Disagree to 5 = Strongly Agree.

#### 3.2.6 Work alienation

The level of work alienation among hotel employees, on the other hand, was measured using the unidimensional, eight-item “Work Alienation Scale” developed by Nair and Vohra (2009). This scale assesses key aspects of alienation, such as feelings of meaninglessness, powerlessness, and detachment toward one's job.

### 3.3 Data analysis

**Figure 1:**
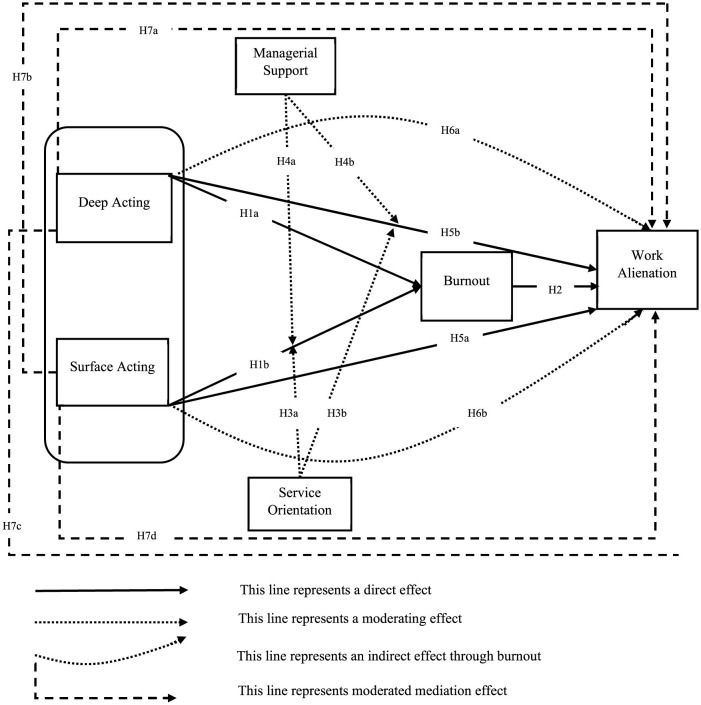
Research model.

Partial least squares structural equation modeling (PLS-SEM) was employed in this study to test the research model as presented in Figure 1. The model consists of six constructs, with one mediating effect and two moderating effects examined among them. Data analysis was conducted using SmartPLS version 4.1.0.9. The PLS-SEM results were evaluated in two phases (Hair et al., 2022). The measurement model was assessed in the first phase. The assessment concerns the validity and reliability of the constructs. In the second phase, the structural model was evaluated, which includes the testing of the predictive power of the model and the hypotheses regarding the relationships among the constructs. Before proceeding to the data analysis phase, the dataset underwent a detailed preliminary review.

First, a missing value analysis was conducted. Following the procedure recommended by Hair et al. (2017), data from 34 participants with missing values were removed from the dataset. Next, skewness and kurtosis values were calculated to examine the distributional characteristics of the data. Skewness values ranged between −0.382 and 0.294, while kurtosis values ranged between −1.339 and −0.476. These values remained below the threshold of 2.00 specified by Kline (2011), indicating that the data did not deviate significantly from normality and reasonably satisfied the assumption of normal distribution. Finally, the cleaned and preprocessed data from 1,252 participants were used for the final analysis.

## 4 Findings

### 4.1 Demographic findings

The participants in the study consisted of a total of 1,252 frontline employees. Demographic characteristics of the participants are presented in detail in Table 1. According to the findings, 44% of the participants were female and 56% were male. In terms of marital status, the majority of participants (59%) were single, while 41% were married. Regarding age distribution, most participants (30%) were between the ages of 25 and 31, followed by those aged 32–38 (27%), 18–24 (24%), 39–45 (11%), and 45 and older (8%). With respect to educational background, the largest proportion of participants held a high school diploma (45%), followed by elementary education (28%), associate degree (18%), bachelor's degree (6%), and master's degree (3%). In terms of departmental distribution, the largest group worked in the food and beverage department (55%), followed by reception (28%), entertainment (11%), and guest relations (6%). As for tenure at their current organization, the majority of employees had between 1 and 3 years of experience (31%). Those with less than 1 year accounted for 24%, followed by 4–6 years (17%), 7–9 years (16%), and 10 years or more (11%).

**Table 1:** Demographic findings (*n* = 1,252).

**Variables**	**Items**	***n***	**Percentage (%)**
Gender	Female	549	44
	Male	703	56
Marital Status	Married	513	41
	Single	739	59
Age	18–24	299	24
	25–31	376	30
	32–38	338	27
	39–45	138	11
	45 and above	101	8
Education	Elementary	351	28
	High school	563	45
	Associate degree	225	18
	Bachelor's	71	6
	Master's	42	3
Department	Food and beverage	685	55
	Reception	348	28
	Customer relations	77	6
	Entertainment	142	11
Tenure	Less than a year	304	24
	1–3 years	385	31
	4–6 years	218	17
	7–9 years	205	16
	10 years and above	140	11

### 4.2 Measurement model

In the study, the outer model was evaluated first, followed by an assessment of the inner model. The outer model analysis focused on reliability and construct validity criteria in detail. The results of the outer model are presented in Table 2. During the examination of the outer model, outer loadings were analyzed first. According to the findings, all outer loadings exceeded 0.70 and were statistically significant at the 0.05 level, indicating acceptable item reliability. Additionally, the composite reliability (CR) and Cronbach's alpha coefficients for each construct were above 0.70, demonstrating internal consistency among the constructs. Furthermore, all constructs had average variance extracted (AVE) values above 0.50, confirming convergent validity (Hair et al., 2019). Two main approaches were used to assess discriminant validity (Table 3). First, following the Fornell and Larcker (1981) criterion, the square root of each construct's AVE was found to be greater than its correlations with other constructs, supporting discriminant validity. Second, the Heterotrait-Monotrait ratio (HTMT) was evaluated. HTMT values were below 0.85, further confirming discriminant validity (Henseler et al., 2015). In conclusion, the outer model analysis indicated that the reliability and validity criteria were met, with item reliability, internal consistency, convergent validity, and discriminant validity all at satisfactory levels.

**Table 2:** Result of outer model.

**Construct**	**Items**	**Outer loadings**	***t* values**	**α**	**CR**	**AVE**
Deep acting	I make an effort to genuinely feel the emotions I am required to display	0.911	130.873^*^	0.900	0.938	0.766
	I try my best to actually feel the emotions I need to show to guests	0.783	47.280^*^			
	I put intense effort into feeling the emotions I must show to guests	0.894	106.505^*^			
	I strive to truly feel the emotions I am obliged to display to guests	0.907	118.967^*^			
Surface acting	My smile is often not sincere	0.901	175.744^*^	0.936	0.938	0.759
	I fake the emotions I show when dealing with customers	0.804	72.794^*^			
	I feel as if I have a split personality when interacting with customers because I act not like myself at all	0.850	103.903^*^			
	I put on an act in order to deal with customers in an appropriate way	0.879	132.208^*^			
	I put on a mask in order to express the right emotions for my job	0.894	142.266^*^			
	I display emotions that I am not actually feeling	0.895	145.305^*^			
Burnout	How often do you feel tired?	0.911	153.651^*^	0.956	0.956	0.791
	How often are you physically exhausted?	0.827	105.409^*^			
	How often are you emotionally exhausted?	0.888	148.673^*^			
	How often do you think: “I can't take it anymore”?	0.891	171.389^*^			
	How often do you feel worn out?	0.892	150.363^*^			
	How often do you feel weak and susceptible to illness?	0.898	157.281^*^			
	How often do you feel tired?	0.916	197.184^*^			
Service orientation	I voluntarily assist customers even if it means going beyond job requirement	0.895	144.830^*^	0.924	0.925	0.767
	I help customers with problems beyond what is expected or required	0.835	87.339^*^			
	I often go above and beyond the call of duty when serving customers	0.875	116.535^*^			
	I willingly go out of his/her way to make a customer satisfied	0.871	119.165^*^			
	I frequently go out the way to help a customer	0.902	153.483^*^			
Managerial support	My supervisor will help employees solve work-related problems	0.924	186.167^*^	0.952	0.955	0.807
	My supervisor encourages employees to develop new skills	0.844	102.842^*^			
	My supervisor will praise good work performance	0.890	132.642^*^			
	My supervisor cares about employees' feelings and thoughts	0.880	125.972^*^			
	My supervisor cares about my overall satisfaction with the job	0.918	166.294^*^			
	My supervisor will seriously consider my goals and values	0.932	211.909^*^			
Work alienation	I do not enjoy my work; I just put in my time to get paid	0.888	132.012^*^	0.954	0.955	0.756
	Facing my daily tasks is a painful and boring experience	0.821	86.133^*^			
	Work to me is more like a chore or burden	0.845	94.878^*^			
	I feel estranged/disconnected from myself	0.852	95.170^*^			
	I often wish I were doing something else	0.872	122.125^*^			
	Over the years I have become disillusioned about my work	0.879	136.781^*^			
	I do not feel like putting in my best effort at work	0.894	150.689^*^			
	I do not feel connected to the events in my workplace	0.903	148.449^*^			

α = Cronbach's Alpha. ^*^p < 0.001.

**Table 3:** Results of discriminant validity.

**Fornell-Larcker**	**D-Act**	**S-Act**	**BRNT**	**Man-Supp**	**Serv-Orien**	**W-Alien**
D-Act	**0.875**					
S-Act	−0.136	**0.871**				
BRNT	−0.166	0.604	**0.890**			
Man-Supp	0.500	−0.139	−0.554	**0.898**		
Serv-Orien	0.566	−0.282	−0.564	0.682	**0.876**	
W-Alien	−0.264	0.527	0.777	−0.530	−0.571	**0.870**
**HTMT**						
1. Deep acting						
2. Surface acting	0.140					
3. Burnout	0.169	0.638				
4. Managerial support	0.541	0.146	0.579			
5. Service orientation	0.616	0.302	0.600	0.728		
6. Work alienation	0.274	0.556	0.813	0.555	0.609	

The diagonal values in bold are square roots of AVEs. BRNT, Burnout; D-Act, Deep acting; Man-Supp, Managerial support; Serv-Orien, Service orientation; S-Act, Surface acting; W-Alien, Work alienation.

### 4.3 . Inner model

Following the confirmation of validity in the outer model analysis, the inner structure of the model was examined, and analyses were conducted to test the research hypotheses. In the evaluation process of the inner model results, the first step was to examine whether multicollinearity existed among the variables (Hair et al., 2022). As shown in Table 4, all VIF values being below 5 indicates that there is no multicollinearity problem in the model. To test the hypotheses, the bootstrapping method, one of the resampling techniques, was used, with the number of bootstrap subsamples set to 5,000. To evaluate the model's overall fit, the standardized root mean square residual (SRMR) metric was considered. The SRMR value was calculated as 0.032, which is well below the 0.08 threshold suggested by Henseler et al. (2016), indicating that the model exhibits an acceptable level of fit. In the evaluation of the hypotheses, the model's explanatory and predictive power were assessed by taking into account path coefficients (β), impact size (*f*^2^), the determination coefficient (*R*^2^) and *Q*^2^ values.

**Table 4:** Result of structural model.

**Relation**	**Path coefficients**	***t* values**	***p* values**	**Bias corrected**		**VIF**	***R* ^2^**	***f* ^2^**	***Q* ^2^**
				**LB**	**UB**				
D-Act → BRNT	−0.087	3.897	0.000	−0.131	−0.045	1.019	0.373	0.012	0.370
S-Act → BRNT	0.592	25.680	0.000	0.547	0.636	1.019		0.549	
BRNT → W-Alien	0.775	59.993	0.000	0.751	0.801	1.000	0.603	1.521	0.294

BRNT, Burnout; D-Act, Deep acting; S-Act, Surface acting; W-Alien; Work alienation.

According to the structural model findings presented in Table 4, three hypotheses were tested, each yielding statistically significant results. The variables Deep Acting and Surface Acting explain 37.3% of the variance in burnout (*R*^2^ = 0.373). Additionally, the *Q*^2^ value was calculated as 0.370, indicating good predictive relevance of the explanatory variables. The effect of Deep Acting on burnout (D-Act → BRNT) is negative and statistically significant (β = −0.087, *t* = 3.897, *p* < 0.001). The impact size of the relation in question was calculated to be *f*^2^ = 0.012. Although the impact size is small, the results support hypothesis H1a. Surface Acting emerged as the strongest predictor of burnout (S-Act → BRNT) with a strong and positive relationship (β = 0.592, *t* = 25.680, *p* < 0.001). The impact size (*f*^2^ = 0.549) indicates that surface acting has a substantial effect on burnout. Based on these findings, hypothesis H1b is supported as well. Moreover, the effect of burnout on work alienation was found to be strong and statistically significant (β = 0.775, *t* = 59.993, *p* < 0.001). The coefficient of determination (R^2^) for work alienation was 0.603, indicating that burnout explains 60% of the variance in work alienation. The impact size (*f*^2^ = 1.521) reflects a strong predictive power, confirming that burnout is a key determinant of work alienation. Additionally, the *Q*^2^ value of 0.294 suggests that the model has high explanatory and predictive power. Based on these findings, hypothesis H2 is also supported. Table 5 below presents a detailed version of the model, exploring the factors that may affect burnout and their interactions.

**Table 5:** Results of moderation analysis.

**Relation**	**Path coefficients**	***t* values**	**Confidence intervals**		***R* ^2^**	***f* ^2^**	***Q* ^2^**
			**Lower**	**Upper**			
D-Act → BRNT	0.136	7.419	0.098	0.170	0.74	0.044	0.741
S-Act → BRNT	0.475	29.522	0.443	0.507		0.770	
Man-Supp → BRNT	−0.431	19.362	−0.473	−0.387		0.371	
Serv-Orien → BRNT	−0.190	7.700	−0.239	−0.141		0.062	
Serv-Orien × S-Act → BRNT	−0.136	5.833	−0.179	−0.088		0.056	
Man-Supp × D-Act → BRNT	−0.179	9.462	−0.216	−0.141		0.072	
Man-Supp × S-Act → BRNT	−0.152	7.249	−0.194	−0.112		0.049	
Serv-Orien × D-Act → BRNT	−0.063	2.914	−0.105	−0.020		0.007	

BRNT, Burnout; D-Act, Deep acting; Man-Supp, Managerial support; Serv-Orien, Service orientation; S-Act, Surface acting; W-Alien, Work alienation.

Table 5 analyzes the role of managerial support and service orientation, as well as their interactions with emotional labor constructs (D-Act/S-Act) in predicting burnout. Together, D-Act, S-Act, Man-Supp, Serv-Orien and their interactions explain 74% of the variance in burnout (*R*^2^ = 0.74). In addition, the *Q*^2^ value of 0.741 indicates that the model's predictive performance is quite strong, confirming its strong forecasting power. Table 5 shows that the direct effect of deep acting on burnout (D-Act → BRNT) is positive (β = 0.136, *t* = 7.419). The moderation analysis reveals that the negative effect typically associated with deep acting on burnout shifts in a positive direction under certain conditions. This suggests that deep acting does not always reduce burnout directly and, in some contexts, may even contribute to increased levels of burnout. On the other hand, surface acting remains one of the strongest predictors (β = 0.475, *t* = 29.522) and increases burnout levels. The findings in Table 5 also indicate that Man-Supp and Serv-Orien factors may also moderate this relationship. The direct effect of managerial support on burnout (Man-Supp → BRNT) was found to be strong and negative (β = −0.431, *t* = 19.362). This indicates that managerial support serves as a direct protective factor against burnout. Similarly, service orientation (Serv-Orien → BRNT) appears as a mitigating factor in relation to burnout (β = −0.190, *t* = 7.700). These results demonstrate that both managerial support and service orientation significantly reduce employees' levels of burnout.

According to the results presented in Table 5, the interaction effects of Man-Supp and Serv-Orien partially buffer the amplifying effects of emotional labor strategies (D-Act/S-Act) on burnout. The interaction between Serv-Orien × S-Act (β = −0.136, *t* = 5.833) indicates that employees with high service orientation are able to partially mitigate the negative impact of surface acting on burnout (H3a). The interaction of Man-Supp × D-Act (β = −0.179, *t* = 9.462) shows that managerial support buffers the positive effect of deep acting on burnout and plays a significant moderating role in reducing employees' burnout levels (H4b). Similarly, the interaction of Man-Supp × S-Act (β = −0.152, *t* = 7.249) demonstrates that the presence of managerial support partially reduces the burnout-inducing effect of surface acting (H4a). Lastly, the interaction of Serv-Orien × D-Act (β = −0.063, *t* = 2.914) suggests that service-oriented employees can use deep acting more effectively, though the moderating effect remains relatively weak (H3b). These findings support hypotheses H3a, H3b, H4a, and H4b. The results presented in Table 5 indicate that both organizational factors such as managerial support and individual traits such as service orientation interact with emotional labor strategies to reduce burnout.

Table 6 presents the results of the mediation analysis examining the effects of deep and surface acting on work alienation through burnout. The total effect of deep acting on work alienation (D-Act → W-Alien) was found to be negative and statistically significant (β = −0.196, *t* = 8.863). The total effect of surface acting on work alienation (S-Act → W-Alien) was found to be positive and statistically significant (β = 0.500, *t* = 23.491, *p* < 0.05). These results support hypotheses H5 and H5b. The indirect effect of deep acting on work alienation through burnout (D-Act → BRNT → W-Alien) was also statistically significant (β = −0.060, *t* = 3.752, *p* < 0.05). This indicates that deep acting reduces work alienation in part by lowering burnout. This finding supports the presence of partial mediation by burnout in the relationship between deep acting and work alienation. Thus, it supports hypothesis H6a. The indirect effect of surface acting on work alienation through burnout (S-Act → BRNT → W-Alien) was statistically significant as well (β = 0.417, *t* = 19.884, *p* < 0.001). This finding shows that surface acting increases work alienation substantially through burnout. Accordingly, hypothesis H6b is also supported at the level of partial mediation.

**Table 6:** Results of mediation analysis.

**Relation**	**Indirect effect**	***t***	***p***	**Direct effect**	***t***	***p***	**Total effect**	***t***	***p***	**Type of mediation**
D-Act → BRNT → W-Alien	−0.060	3.752^*^	0.00	–	–	–	−0.196	8.863^*^	0.00	Partial mediation
D-Act → W-Alien	–	–	–	−0.136	9.128^*^					
S-Act → BRNT → W-Alien	0.417	19.884^*^	0.00	–	–	–	0.500	23.491^*^	0.00	Partial mediation
S-Act → W-Alien	–	–	–	0.083	3.912^*^					

BRNT, Burnout; D-Act, Deep acting; S-Act, Surface acting; W-Alien, Work alienation. ^*^p < 0.001.

**Figure 2:**
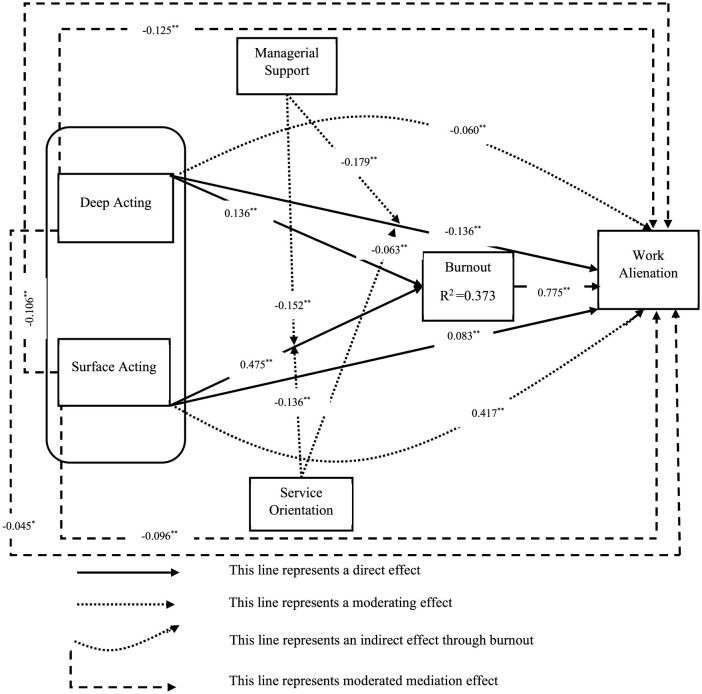
Result of hypothesized moderated mediation. **p < 0.001; *p < 0.01.

Table 7 presents the results of the moderated mediation analysis. This analysis tested how managerial support and service orientation function as moderators in the indirect effect of emotional labor strategies (D-Act/S-Act) on work alienation through burnout. The findings indicate that deep acting affects work alienation positively (D-Act → BRNT → W-Alien) through burnout (β = 0.097; *t* = 7.107; *p* < 0.001). While the results of the mediation analysis in Table 6 reveal that deep acting generally tends to reduce work alienation, this apparent strategy does not always provide a “fully protective effect.” The findings also emphasize the importance of moderating (regulatory) variables. The interaction between deep acting and managerial support (Man-Supp × D-Act) significantly reduced burnout (β = −0.125; *t* = 9.264; *p* < 0.001) and, in turn, work alienation. This shows that managerial support mitigates the emotional costs associated with deep acting. Similarly, the interaction between service orientation and deep acting (Serv-Orien × D-Act) partially decreases burnout (β = −0.045; *t* = 2.954; *p* < 0.05) and reduces work alienation in addition. Surface acting was found to increase work alienation through burnout. The emotional dissonance that results from expressing inauthentic emotions contributes to intense burnout and subsequently leads to alienation. However, the interaction between surface acting and managerial support (Man-Supp × S-Act) leads to a negative effect on work alienation through burnout (β = −0.106; *t* = 7.008; *p* < 0.001). Similarly, the interaction between surface acting and service orientation (Serv-Orien × S-Act) reduces work alienation through burnout (β = −0.096; *t* = 5.749; *p* < 0.001). The structural model of the study is presented in Figure 2. These results show that both managerial support and service orientation have the potential to mitigate the negative emotional consequences of surface acting. Overall, the findings support hypotheses H7a, H7b, H7c, and H7d.

**Table 7:** Results of moderated mediation analysis.

**Relation**	**Specific indirect effect**	***t***	***p***
D-Act → BRNT → W-Alien	0.097	7.107	0.000
S-Act → BRNT → W-Alien	0.334	21.286	0.000
Man-Supp → BRNT → W-Alien	−0.303	17.375	0.000
Serv-Orien → BRNT → W-Alien	−0.135	7.532	0.000
Man-Supp × S-Act → BRNT → W-Alien	−0.106	7.008	0.000
Man-Supp × D-Act → BRNT → W-Alien	−0.125	9.264	0.000
Serv-Orien × D-Act → BRNT → W-Alien	−0.045	2.954	0.003
Serv-Orien × S-Act → BRNT → W-Alien	−0.096	5.749	0.000

BRNT, Burnout; D-Act, Deep acting; Man-Supp, Managerial support; Serv-Orien, Service orientation; S-Act, Surface acting; W-Alien, Work alienation.

## 5 Discussion

Conducted with the participation of frontline employees in the hospitality sector, this study aimed to examine, within an integrated model framework, the mediating role of burnout in the relationship between emotional labor strategies (deep acting and surface acting) and work alienation, as well as the moderating effects of service orientation and managerial support in this process. The overall findings reveal the complex relationship between emotional labor and work alienation. First of all, the two emotional labor strategies (deep acting and surface acting) were found to have opposite effects on burnout and work alienation. Surface acting was found to increase both burnout and work alienation, whereas deep acting mitigated these negative outcomes. More importantly, burnout was identified to have a partial mediating role in the relationship between both emotional labor strategies and work alienation.

Another critical contribution of the study lies in signifying that this negative spiral can be interrupted. Findings revealed that both service orientation, as an individual resource, and managerial support, as an organizational resource, significantly buffered the detrimental effects of emotional labor on burnout and, consequently, on work alienation. Service orientation and managerial support functioned as protective buffers in the relationship between emotional labor and burnout, reducing the exhausting impact of surface acting while reinforcing the protective impact of deep acting. Additionally, these individual and organizational protective factors also exhibited moderating effects on the mediation path of burnout, offering valuable insights regarding potential intervention points. This study further provides an empirical roadmap for identifying which individual and organizational resources should be strengthened to mitigate the negative consequences by emphasizing the importance of emotional labor management for the psychological wellbeing and organizational attitudes of service employees.

### 5.1 Theoretical implications

The findings of this study contribute to the existing literature in three main ways. First, the results demonstrate that employees experience different outcomes in terms of burnout and, through burnout, work alienation depending on the emotional labor strategy they adopt. The findings reveal that surface acting increases burnout and, through burnout, work alienation, whereas deep acting reduces both. In this way, the prevailing view that emotional labor is an inherently stressful process with negative outcomes (Hochschild, 1983; Grandey, 2000) is differentiated based on specific emotional labor strategies (surface acting and deep acting). The study elucidates which emotional labor strategies, under organizational display rules, become resource-depleting processes. In surface acting, employees must suppress genuine emotions while pretending to feel emotions they do not actually experience. When employees fail to cope directly with the stress arising from displaying unfelt emotions (Hochschild, 1983) this emotional discrepancy accelerates resource depletion and adds to burnout. Burnout, in turn, creates a psychological condition that distances employees from their work, thereby reinforcing work alienation (Akar, 2018; Conway et al., 2018; Inandi and Büyüközkan, 2022; Khan et al., 2019; Öztürk Çiftçi, 2021). Sun et al. (2025) reported that surface acting directly leads employees toward exhaustion by creating emotional dissonance and ultimately strengthens turnover intention. Conversely, deep acting enhances emotional alignment through the internalization and meaning making of displayed emotions, limiting resource erosion and consequently reducing burnout and alienation. This finding highlights the decisive role of contextual factors such as person–role fit and the congruence between display rules and self-image norms. From the perspective of the Conservation of Resources (COR) theory (Hobfoll, 1989), employees strive to protect and enhance their valuable resources. When there is congruence between emotional display rules and self-image, deep acting can enhance emotional coherence and slow down the resource-loss spiral, whereas surface acting accelerates resource loss. In particular, the detrimental effects of surface acting intensify as the discrepancy between self-image and organizational display rules deepens, while the protective effects of deep acting become more evident as this congruence increases. Thus, the findings identify which emotional labor strategies, and under which conditions, transform into processes that deplete resources.

Secondly, drawing on the Job Demands–Resources (JD–R) model, the study incorporates two fundamental moderating variables in the relationship between emotional labor and burnout: service orientation (a personal resource) and managerial support (a job resource). Research on emotional labor has traditionally emphasized that surface acting, in particular, constitutes a major stressor that leads to burnout through emotional dissonance and resource depletion (Amissah et al., 2021; Hur et al., 2013; Jeung et al., 2018; Lv et al., 2012; Satyanarayana and Shanker, 2012; Zhang et al., 2018). The study sought to address the question: “*Which factors* can buffer this negative effect?” Accordingly, the study aimed to contribute to the literature by examining the protective mechanisms of individual (service orientation) and organizational (managerial support) resources. The findings confirm that both factors serve as significant buffers against the detrimental effects of emotional labor on burnout.

It was found in this study that service orientation significantly weakened the effects of both surface acting and deep acting on burnout. This finding supports the positive associations between service orientation and personality traits that cultivates emotional resilience reported by Donavan (1999), Köşker et al. (2019), and Tekin and Kalkan (2017). Employees with a high level of service orientation experience less emotionally draining feelings during service delivery (Bakar et al., 2020), are able to limit the potential negative consequences of surface acting in customer relations (Çoban and Aytemiz Seymen, 2019), and more often employ deep acting to strengthen emotional alignment, which in turn reduces emotional burnout. Similarly, the negative relationship between service orientation and burnout (Yoo et al., 2015) and evidence suggesting that enhancing service orientation may reduce burnout by promoting deep rather than surface acting (Igbojekwe, 2017; Lee et al., 2018; Yang et al., 2020) further support our results. Within this framework, service orientation can be regarded as an important personal resource that lowers the psychological costs generated by emotional labor. Furthermore, perceived managerial support was also found to serve as a strong buffer in the relationship between emotional labor and burnout. The literature provides consistent evidence that managerial support reduces burnout (Blanch and Aluja, 2012; Bektas and Peresadko, 2013; Cheng et al., 2025; Khatatbeh et al., 2020; Qotimah et al., 2021) and influences employees' preferences for deep versus surface acting as well as their sense of job satisfaction (Lee et al., 2012; Hur et al., 2013). Recent studies also point out that managerial support enhances job satisfaction and encourages deep acting, reducing surface acting and burnout (Altaş et al., 2024).

Third, the findings of this study extend the current understanding of emotional labor by revealing the critical moderating roles of personal (service orientation) and organizational (managerial support) resources in the indirect effect of emotional labor strategies on work alienation. The research model demonstrated that both service orientation and managerial support function as moderating (buffering) mechanisms along the deep/surface acting → burnout → work alienation pathway. In particular, managerial support reduces work alienation by lowering burnout, while service orientation produces a similar protective effect by attenuating the influence of emotional labor on burnout. Thus, the mixed findings regarding the negative consequences of emotional labor are reinterpreted within an integrative framework that clarifies, for whom and under which conditions these effects intensify. Within this framework, the results offer a comprehensive explanation of how the adverse outcomes of emotional labor can be effectively buffered by the resources that employees possess and those provided by the organization.

The results obtained from this study advance the JD–R model within the context of emotional labor. The literature has consistently shown that surface acting, due to the suppression of genuine emotions, constitutes a resource-depleting demand (Amissah et al., 2021; Ha et al., 2021; Kurt et al., 2019; Lee, 2019; Lu and Guy, 2019; Sun et al., 2025; Zhang et al., 2018). The findings not only confirm this fundamental relationship but also reveal the mechanisms of two specific resources that break the “demands → burnout → alienation” chain. As a personal resource, service orientation, and as an organizational resource, managerial support, both function as buffers against burnout, reducing the psychological costs of emotional labor and ultimately lowering the risk of work alienation. Job demands such as emotional display rules and intense customer contact require continuous self-regulation, which can trigger burnout through resource depletion (Tummers and Bakker, 2021). Surface acting, in particular, accelerates resource erosion as a result of displaying unfelt emotions and suppressing genuine ones (Yikilmaz et al., 2024; Zhang et al., 2018). Our findings demonstrate that service orientation, as a personal resource, and managerial support, as an organizational resource, both serve buffering functions along this demand–burnout pathway. Service orientation, due to their intrinsic predisposition, operates as a protective mechanism by enabling individuals to cope more effectively with emotional demands and to favor the less resource-depleting deep acting strategy. Managerial support, on the other hand, functions as an external resource that balances employees' emotional load and provides a stabilizing mechanism against strain. The simultaneous confirmation of these two factors within a single model highlights two complementary intervention axes in mitigating the adverse outcomes of emotional labor: recruitment practices emphasizing person–job fit, and management practices centered on supportive leadership.

In conclusion, this study contributes to the literature on the emotional labor—burnout—work alienation relationship by jointly addressing its mechanisms of influence through (1) the parallel buffering effects of personal and organizational resources, (2) the quality of emotional regulation strategies, and (3) the resource dynamics grounded in the JD–R/COR frameworks. In doing so, the study moves beyond explaining “how much” emotional labor exhausts employees to show which employees and under which conditions it does so; which allows for the theoretical definition of boundary conditions and the practical design of targeted managerial tools (improving managerial support, strengthening service orientation).

### 5.2 Practical implications

This research study demonstrates that the ways in which frontline employees in hospitality organizations regulate their emotions (deep vs. surface acting) influence employee wellbeing and provide clear, actionable insights for managing the negative consequences of emotional labor. Given the dynamic nature of the work environment, employees need both personal and organizational resources to cope with emotional demands. The present study identified service orientation and managerial support as critical resources that alleviate the detrimental effects of emotional labor on burnout and work alienation. Within this framework, several practical implications can be derived from the findings.

First of all, service orientation should be considered a critical selection criterion in recruitment and hiring processes. As supported by Donavan (1999), Köşker et al. (2019), and Tekin and Kalkan (2017), individuals with high service orientation exhibit personality traits that foster emotional resilience. Therefore, interviewers should include situational questions that allow candidates to demonstrate their service-oriented attitudes and previous experiences. Second, training programs should be organized to strengthen existing employees' service orientation and emotional resilience. The findings of Bakar et al. (2020) and Yoo et al. (2015) indicate that service orientation reduces emotionally exhausting experiences and is negatively related to burnout. Accordingly, training that cultivates emotional awareness, empathy, and stress management skills can protect employees from the adverse effects of *surface acting* and promote *deep acting*, a more authentic form of emotional engagement. These types of programs can help employees manage their emotional capital as a personal resource.

Upper management should also be encouraged and trained to provide managerial support to their subordinates. First, managers must be aware of the psychological costs associated with emotional labor and ensure that employees have the necessary resources, such as time, recognition, and flexibility, to cope with these costs. Prior studies (Blanch and Aluja, 2012; Bektas and Peresadko, 2013; Khatatbeh et al., 2020) confirm that managerial support directly reduces burnout. Second, managers should be equipped with the skills to observe employees' emotional states and provide constructive emotional feedback. Evidence from Altaş et al. (2024), Cheng et al. (2025), Lee et al. (2012), and Hur et al. (2013) demonstrated that managerial support can influence employees' *deep acting/surface acting* preferences. Managers should, therefore, reinforce employees' self-efficacy by recognizing their achievements and offering positive feedback, while creating a psychologically safe environment that encourages authentic emotional expression.

Managers should recognize emotional labor as a job demand and take proactive measures to counterbalance the resource depletion it causes. Within the framework of the Job Demands–Resources (JD–R) model, resource-draining job demands must be offset by resources such as service orientation and managerial support. In this context, managers may implement measures such as providing “cool-down periods” after emotionally demanding shifts or establishing peer support groups following difficult customer interactions. As pointed out by Zhang et al. (2018) and Yikilmaz et al. (2024), such practices can slow the resource erosion caused by surface acting. Overall, this research study offers an empirically grounded roadmap for managers seeking to design work environments that minimize the psychological costs of emotional labor. The proposed model is based on two complementary strategies: (1) selecting employees with high service orientation during recruitment and (2) fostering supportive managerial practices within organizations. This dual approach is expected to protect employees' psychological wellbeing while simultaneously enhancing service quality.

### 5.3 Limitations and future research direction

Despite the significant contributions of this study, several limitations should be carefully considered and addressed in future research. The sample was limited to frontline roles in the hospitality sector in Türkiye. Because emotional display rules, the intensity of customer contact, and the nature of service processes vary across industries, the generalizability of the findings may be limited. Therefore, multi-sectoral research designs that include diverse service domains such as healthcare, banking, education, retail, and aviation are recommended. The use of data collected from a single country also constrains the examination of cross-cultural variability (e.g., normative expectations, the rigidity of display rules and so on). Future studies incorporating cross-cultural comparative designs would enhance the universality of the findings. This study demonstrated that surface acting can be detrimental while deep acting may be protective depending on contextual factors, and that service orientation and managerial support buffer the relationship between emotional labor and burnout. It is suggested future research explore this buffering mechanism more deeply by testing moderated moderation (i.e., three-way interaction) models. In this context, it would be valuable to investigate whether the protective effects of service orientation and managerial support are themselves contingent on other conditions. For example, person-organization fit (as a third-level moderator) could be tested to determine if it strengthens (or weakens) the buffering capacity of service orientation and managerial support on the emotional labor-burnout relationship. To that end, multilevel models that simultaneously account for individual, team, and organizational levels, as well as conditional indirect effect analyses (moderated mediation), are recommended. Furthermore, experimental studies are also strongly recommended to increase the causal validity of the practical implications. Specifically, testing whether programs designed to strengthen service orientation through selection, placement, and training, as well as supportive leadership practices, can enhance the use of deep acting and reduce surface acting and burnout would provide a valuable contribution to the field.

## Data Availability

The raw data supporting the conclusions of this article will be made available by the authors, without undue reservation.
